# Grassroots Autonomy: A Laypersons' Perspective on Autonomy

**DOI:** 10.3389/fpsyg.2022.871797

**Published:** 2022-04-07

**Authors:** Elli Zey, Sabine Windmann

**Affiliations:** Cognitive Psychology, Department of Psychology, Goethe University Frankfurt, Frankfurt am Main, Germany

**Keywords:** autonomy, bottom-up process, dignity, independence, morality, self-awareness, unconventionality

## Abstract

In the age of artificial intelligence, the common interest in human autonomy is experiencing a revival. Autonomy has formerly and mostly been investigated from a theoretical scientific perspective, in which scholars from various disciplines have linked autonomy with the concepts of dignity, independence from others, morality, self-awareness, and unconventionality. In a series of three semi-qualitative, preregistered online studies (total *N* = 505), we investigated laypersons' understanding of autonomy with a bottom-up procedure to find out how far lay intuition is consistent with scientific theory. First, in Study 1, participants (*n* = 222) provided us with at least three and up to 10 examples of autonomous behaviors, for a total of 807 meaningful examples. With the help of blinded research assistants, we sorted the obtained examples into categories, from which we generated 34 representative items for the following studies. Next, in Study 2, we asked a new sample of participants (*n* = 108) to rate the degree of autonomy reflected in each of these 34 items. Last, we presented the five highest-rated and the five lowest-rated items to the participants of Study 3 (*n* = 175), whom we asked to evaluate how strongly they represented the components of autonomy: dignity, independence from others, morality, self-awareness, and unconventionality. We identified that dignity, independence from others, morality, and self-awareness significantly distinguished between high- and low-autonomy items, implying that high autonomy items were rated higher on dignity, independence from others, morality, and self-awareness than low autonomy items, but unconventionality did not. Our findings contribute to both our understanding of autonomous behaviors and connecting lay intuition with scientific theory.

## Introduction

Autonomy (Greek αuτóνoμoς: “auto” means self and “nomos” means law) is a highly discussed concept in philosophy, education, psychology, medicine, rehabilitation, law, artificial intelligence, and other applied sciences. It is seen as an essential component of human life and a key democratic requirement, for example in Rousseau's political philosophy (Cohen, 1986). But despite its popularity, the meaning of the term is vague (Anderson et al., 1994), and regardless of its frequent use, there is little communication between scholars and the general public regarding the understanding of the concept. For instance, in constitutional law, autonomy is defined as “the condition in which what one does reflects who one is” (Weinrib, 2019), whereas psychologists say that autonomous individuals “establish in a self-determined fashion their own life goals, criteria for their happy and good lives, and the moral standards, which they rationally decide to pursue to be happy and successful” (Chirkov, 2011, p. 611). Interestingly, both the Greek philosopher Aristotle and the psychological Social Determination Theory (SDT) define autonomy as self-rule or self-government (Ryan and Deci, 2006; Pérez and Ziemke, 2007). These various attempts at defining autonomy show how abstract and difficult it is to operationalize the term (Keenan, 1999). Indeed, several scholars demand specification of the concept of autonomy beyond theory and in the light of real-world implications and usability (Keenan, 1999; Racine et al., 2021). In the past, especially in the psychological literature, the focus lay more on the opposites of autonomy, in connection with conformity, compliance, and the bystander effect (Asch, 1961; Cialdini and Goldstein, 2004; Kundu and Cummins, 2013; Bostyn and Roets, 2017). By contrast, research on dissidence, deviance, or resistance, for which autonomy appears to be a prerequisite, is underrepresented (Swann and Jetten, 2017). Understanding what autonomy means from an applied everyday perspective could aid in setting up psychological surveys and experiments as well as interpreting their outcomes, in addition to improving the communication of its scientific conceptualization to the public.

Reviewing the scientific, philosophical, and psychological literature, we find five components that are repeatedly linked with autonomy. The first is dignity, referring to the most abstract principle regulating the relationship between the rulers and the ruled. Dignity is often equated with the concept of autonomy (Weinrib, 2019). It connects with autonomy in the domains of constitutional law and human rights (Sensen, 2011; Mahlmann, 2012), but also in health care and nursing (Fisher and Oransky, 2008; Delmar, 2013). In philosophy, the conception of autonomy is substantially influenced by Immanuel Kant (May, 1994; Taylor, 2005). According to Kant, a person's dignity emerges from being their moral lawgiver, i.e., from being autonomous (Kant, 1870). This standpoint was shared in psychology (Dworkin, 1988; Erikson, 1998) and was expanded to including the concept of individual autonomy, reflecting an esteemed trait of human beings as the source of human dignity (Racine et al., 2021). Therefore, we propose dignity as one component of autonomy in our study.

Kant defines autonomy as the property by which it is a law to itself, independent of any property of the objects of volition (Kant, 1870). This means that a person with an autonomous character can self-rule independently of any external determination. A similar way of thinking is shared by some developmental psychologists: for Piaget (1983), an individual is “morally” autonomous when decisions and actions are independent of any external influences, especially of adult authority. Others define autonomy directly as resistance against authoritarian and normative influences (Kohlberg, 1981; May, 1994; Erikson, 1998). From this view, acting autonomously requires the ability to decide and act independently of others, whether those others are one's parents in childhood, other authority figures, peers, or merely well-established social norms. To conclude, we suggest *independence from others* as the second essential component of autonomy (Dworkin, 1988).

*Self-awareness* is often discussed in relation to autonomy (Bekker, 1993; Bekker and van Assen, 2008; Pauen and Welzer, 2015; Moleiro et al., 2017). Being self-aware means awareness of one's own opinions, wishes, and needs. Similarly, the Aristotelian concept of autonomy relies on “self-regulation” and is shared by modern psychologists: Ryan and Deci (2006, p. 101860) define autonomy as “a sense of initiative and ownership in one's actions. It is supported by experiences of being externally controlled, whether by rewards or punishments.” They also advocate a proactive and reflective conception of autonomy, one that is based on self-regulatory processes involved in initiating, controlling, and evaluating one's decisions and actions (Swann and Jetten, 2017; Ryan and Deci, 2020). Racine et al. (2021) also argue that the ability to regulate attention, emotions, and behavior is an invaluable component of autonomy since, without it, individuals merely react in the moment instead of taking long-term goals and values into consideration. Thus, autonomous individuals control their development and determine the course of their lives while monitoring the costs and benefits of their choices (Oshana, 2006). In summary, we consider *self-awareness*, in the sense of being aware of one's own opinions, wishes, and needs, as the third component of autonomy.

Kant's foremost statement on autonomy is the term moral autonomy (Kant, 1870). *Morality* displays what is the “right” or “wrong” way in human interaction, for example, being just to others or being unjust (Ellemers et al., 2019). Some scholars value autonomy as the right of individuals to act and decide freely as long as they do not violate the rights of other humans (Dworkin, 1988; Racine et al., 2021). Some also believe that only by acting autonomously do people form their moral standards (Chirkov, 2011). A morally autonomous person reflects on moral principles and critically examines them before approving them (Oshana, 2006). However, although the link between autonomy and morality appears to be evident in theory, there is still a need for specification in empirical research. Taken together, we advocate *morality* as the fourth component of autonomy.

Other conceptions contrast autonomy with norm-oriented thinking and acting. Warren and Campbell (2014) define extreme autonomy as completely ignoring typical conventions and not acting on them. Likewise, Kohlberg et al. (1983) third and highest level of moral development is called the post-conventional level, meaning being unbound by norms and conventions. On this level, the value of ideas and behaviors is no longer predefined by objective principles, social conventions, or subjective feelings and perspectives (Shweder et al., 1990). Such *unconventionality* has empirically been found to predict winding, autonomous career paths (Schwaba et al., 2019). Last, during an epoch of widespread rebellion of students against society's establishment in many Western countries, a study at UCBerkeley run in the 1960s reported a non-conventional, so-called subcultural group to express a significantly higher need for autonomy than a random college student sample (Whittaker and Watts, 1967). Therefore, we suggest *unconventionality* as the fifth component of autonomy.

In summary, social science scholars, mostly philosophers and psychologists, proposed autonomy be defined by dignity, independence from others, morality, self-awareness, and unconventionality. We use these five components (in the preregistration referred to as “criteria”) for our investigation into whether, and to what degree, this scientific perspective corresponds with the understanding of laypersons. In Study 1, we used a qualitative approach, gathering examples of autonomous behaviors from laypersons, which we then categorized systematically with the help of naive research assistants. In Study 2, we asked new participants to rate the categorized behaviors concerning how autonomous they found them. Finally, in Study 3, we tested with yet another sample of participants whether the five behavioral categories rated highest in autonomy produced higher ratings of the components than the five behavioral categories rated lowest in autonomy. We also expected dignity, independence from others, morality, self-awareness, and unconventionality to be moderately inter-correlated (around 0.40).

All three studies were conducted as online surveys, were set up with the SoSci survey tool (Leiner, 2019), and were preregistered before the collection of data (Zey and Windmann, 2020). Written informed consent was obtained in all studies, and the research project was approved by the ethics committee of Goethe University Frankfurt (Reference number: 2019-49, Oct 20th, 2019). Data analyses for all three studies were carried out in R4.1.2 (R Core Team, 2021) using RStudio (RStudio Team, 2021) and the packages *car* (Fox and Weisberg, 2019), *corrgram* (Wright, 2021), *descr* (Enzmann et al., 2021), *dplyr* (Wickham et al., 2021), *ez* (Lawrence, 2016), *ggplot2* (Wickham, 2016), *psych* (Revelle, 2021), *reshape2* (Wickham, 2007), *rstatix* (Kassambara, 2021), *see* (Lüdecke et al., 2021), and *tidyr* (Wickham, 2021). All data and scripts can accessed online *via* the Open Science Framework (Zey and Windmann, 2020).

## Study 1: Laypersons' Examples of Autonomy

### Method

#### Sample

Following our preregistration, we recruited *N* = 222 fully completed online questionnaires *via* social media and our department's homepage. We assessed age (*M* = 34.58, *SD* = 14.61, ranging from 19 to 82 years), education (49.10% university or college degree, 36.94% A-levels, 7.21% trained profession, 4.96% secondary school certificate, 1% school-leaving certificate, and 1% no finished degree), and gender identification (142 females, 70 males, 5 diverse, 5 not specified) (Bekker and van Assen, 2006). Participants completed the questionnaire in *M* = 7.18 min and received no compensation for participating.

#### Materials and Procedure

We asked participants to list at least three and up to 10 examples of autonomous behaviors, asking “What do you consider to be examples of autonomous (self-determined) behaviors?” We obtained a total of 859 examples. Before categorizing, we eliminated 21 examples (1%) for having no meaning (e.g., “xxx”), 23 examples (1%) for paraphrasing core parts of the instruction (e.g., “self-determined”), and 7 examples (<1%) for containing the exact paraphrasing of one of the components used in Study 3 (e.g., “independence”). With the help of two assistants who were blind to the hypothesis of the study and worked independently of one another, we sorted the remaining 807 examples into categories. They clustered examples with the same or very similar meaning (e.g., “healthy eating” and “good nutrition”) into one category. In the end, a third mediating assistant helped to discuss and resolve diverging decisions.

We then defined the minimum size of eight examples per category (~1% of the total), a change from the preregistration, where we had specified a minimum size of two examples per category. Reviewing the materials, we found that a minimum of two examples would have resulted in quite a high number of unequally sized categories. Thus, we dropped 54 examples that were either unique or formed categories with fewer than eight examples (e.g., “planting a tree”). We found 28 singular examples that did not match any other examples (e.g., “giving a talk”) and therefore could not be categorized.

In summary, based on the assistants' categorizations, we sorted 725 examples into 34 categories. See the Open Science Framework project (Zey and Windmann, 2020) for the complete list of unedited responses and all steps of categorization and editing.

Additionally, in all three studies, we assessed 16 items of the horizontal/vertical and individualistic/collectivist orientation short scales (Priestley et al., 2020), as well as marital status, religion, and female rights for other research purposes; these data are not relevant for the present research.

### Results and Discussion

An average of 3.9 responses per participant were taken and categorized into the 34 categories presented in Table 1. In Study 2, we proceeded to ask laypersons as to how autonomous they rate each of these items.

**Table 1:** Frequencies of the 34 edited categories of examples of autonomy behaviors obtained in Study 1 (*N* = 222), and mean autonomy ratings of Study 2 (*N* = 108) in ascending order.

	**Study 1**	**Study 2**	**Study 2, female sub-sample**	**Study 2, male sub-sample**
**Item description (English translation)**	**Frequencies of mentions per category**	***M (SD)***	***M (SD)*** ***n* = 83**	***M (SD)*** ***n* = 24**
Acting contrary to societal expectations and laws	23	3.34 (1.18)	3.30 (1.16)	3.46 (1.28)
Designing working conditions	23	3.46 (1.00)	3.53 (0.98)	3.17 (1.01)
Shaping one's living situation	27	3.55 (1.03)	3.64 (1.04)	3.25 (0.94)
Travel	21	3.56 (1.18)	3.59 (1.12)	3.38 (1.38)
Acting uninfluenced by external factors	31	3.60 (1.16)	3.61 (1.09)	3.50 (1.38)
Shopping and consuming the way one likes it	10	3.69 (1.05)	3.66 (1.05)	3.79 (1.10)
Shaping one's educational path	34	3.78 (1.08)	3.81 (1.12)	3.67 (0.96)
Taking care of oneself financially	19	3.79 (1.06)	3.81 (1.01)	3.71 (1.27)
Positioning oneself politically	36	3.79 (1.12)	3.78 (1.12)	3.92 (1.02)
Determining time schedule and daily schedule	29	3.79 (0.95)	3.82 (0.95)	3.71 (0.95)
Realizing life plan	17	3.83 (0.90)	3.84 (0.92)	3.83 (0.87)
Feeling what one needs	10	3.85 (1.01)	3.92 (1.00)	3.67 (1.05)
Eating, drinking, sleeping, etc., when and how one wants to	33	3.86 (1.11)	3.86 (1.09)	3.83 (1.17)
Allowing irreversible changes to be made to one's body	11	3.86 (1.19)	3.86 (1.23)	3.92 (1.06)
Being mobile and getting around	14	3.87 (0.99)	3.93 (0.92)	3.71 (1.20)
Deciding about expenses and investments	10	3.90 (1.03)	3.87 (1.02)	4.08 (1.02)
Saying no and setting limits	15	3.92 (1.09)	3.93 (1.06)	4.00 (1.10)
Being creative	9	3.94 (1.16)	3.99 (1.11)	3.79 (1.35)
Deciding about love and sexuality	13	3.96 (1.13)	3.98 (1.12)	3.96 (1.20)
Contraception and family planning	11	3.98 (1.08)	3.99 (1.02)	4.00 (1.29)
Being caring about one's own needs	18	3.98 (0.95)	4.00 (1.00)	3.92 (0.78)
Spending free time alone	10	4.01 (1.11)	4.05 (1.11)	3.88 (1.12)
Freely practicing religion and spirituality	13	4.05 (1.05)	4.11 (0.98)	3.79 (1.28)
Developing personality freely	12	4.05 (0.96)	4.10 (0.96)	3.88 (0.99)
Determining clothing style	18	4.06 (0.97)	4.06 (0.92)	4.04 (1.16)
Asserting one's own goals	12	4.06 (0.89)	4.07 (0.89)	4.00 (0.88)
Choosing a profession	40	4.07 (0.98)	4.07 (0.95)	4.04 (1.12)
Expressing opinions	31	4.09 (0.95)	4.13 (0.95)	3.92 (0.97)
Organizing free time	43	4.11 (0.92)	4.12 (0.85)	4.12 (1.15)
Determining with whom one surrounds oneself with	25	4.18 (0.86)	4.19 (0.88)	4.12 (0.85)
Deciding for oneself	46	4.29 (0.88)	4.31 (0.59)	4.25 (0.90)
Thinking critically and questioning	18	4.31 (0.93)	4.29 (0.90)	4.46 (1.02)
Staying true to oneself	16	4.31 (0.88)	4.36 (0.89)	4.17 (0.82)
Choosing partners	27	4.33 (0.90)	4.31 (0.91)	4.38 (0.88)

## Study 2: Ranking Autonomous Acts

### Method

#### Sample

We recruited a new sample *via* social media and collected complete data sets from *N* = 114 participants. As preregistered, we excluded participants for not answering the control question correctly (*n* = 6), leading to *N* = 108 participants. Participants reported ages (*M* = 26.33, *SD* = 8.54) ranging from 19 to 56, education (37.04% university or college degree, 51.85% A-levels, 7.41% trained profession, 3.70% secondary school certificate, and no one with no finished degree), and gender identification (83 females, 24 males, 0 diverse, 1 not specified).

#### Materials and Procedure

Participants were asked to rate “how autonomous” each of the 34 categories of behavioral examples generated in Study 1 “is to them” on a five-point Likert scale (1 = “not at all autonomous” to 5 = “completely autonomous”). One attention check item was presented at a randomized position in the list of valid categorical items. Participants completed the questionnaire in *M* = 5.99 min and were not compensated for participating.

### Results and Discussion

On average, the categories of the examples of Study 1, listed in Table 1, were rated quite high in autonomy (*M* = 3.92, *SD* = 0.59, *CI* [3.81, 4.03] on the scale from 1 to 5). The highest-rated five items yielded a mean rating of *M* = 4.28, *SD* = 0.64, *CI* [4.16, 4.40] and the lowest-rated five items a mean rating of *M* = 3.50, *SD* = 0.73, *CI* [3.36, 3.64]. All categories contained between 9 and 46 examples, and each category included an average of 21 examples. The five high-autonomy items (categories) contained on average 26.4 examples per category, whereas the five low-autonomy items contained on average 25 examples per category, so they are quite comparable in size. The frequencies (number of examples per category) and the autonomy ratings also did not correlate, *r*_(34)_ = 0.05, *p* = 0.77, with the autonomy items.

Table 1 also shows mean ratings of female and male participants separately, demonstrating that the ranking of the five highest- and lowest-rated examples for the two groups is nearly identical. Only the categories ranked fifth (“determining with whom one surrounds oneself with”) and sixth in position (“organizing free time”) are interchanged in their order between the entire sample and the male-only sub-sample.

In Study 3, we compared how well the high- and low-autonomy items reflected the components' dignity, self-awareness, independence from others, and unconventionality, from the standpoint of laypersons.

## Study 3: What Characterizes Acts Perceived as Autonomous?

### Method

#### Sample

We recruited a new sample of participants with the help of students who spread the survey *via* social media and personal contacts. Unexpectedly, a much higher number of data sets (*N* = 478) than preregistered (*N* = 175) were collected within only a few days. After excluding *n* = 53 persons taking longer than 1.75 times the median time (*Mdn* = 7.21 min), as preregistered, we still had *N* = 444 data sets. To accord with the sample size in our preregistration, we considered using only the first 175 participants, but these showed a disproportionately large number of women (147 females, and 28 males, 0 diverse). Therefore, we included only the first *n* = 88 women (50%) in the data analysis, alongside the one diverse participant, and recruited more male participants, up to *n* = 86, so that the final distribution was gender-balanced. However, using the entire sample (*N* = 444), we repeated the analysis and found that the result pattern did not differ in any relevant way (see Supplemental Materials).

Thus, we here report the data of *N* = 175 participants (age: *M* = 38.90, *SD* = 11.13, ranging from 20 to 75 years; education: 62.29% university or college degree, 12.57% trained profession, 19.43% A-levels, 4.57% secondary school certificate, 0.57% school-leaving certificate, and 0.57% no finished degree; gender identification: 88 females, 86 males, 1 diverse). Participation (*M* = 7.72 min) was not compensated for.

#### Materials and Procedure

Participants rated the five highest-and five lowest-rated autonomy items (as found in Study 2) regarding “how strongly these stand for” dignity, independence from others, morality, self-awareness, and unconventionality on a five-point Likert scale (e.g., 1 = “not at all self-aware” to 5 = “completely self-aware”).

#### Statistical Analysis

In line with the preregistration, we first conducted a two-factorial ANOVA (Analysis of Variance) with repeated measures (5 components × 2 autonomy levels). Next, we compared high-autonomy vs. low-autonomy examples on each of the five components separately using the Wilcoxon signed-rank test. We adjusted alpha levels with Bonferroni corrections to α_Bonferroni_ = 0.01 and we used Huynh-Feldt corrected *p*-values to account for violations of sphericity (Girden, 1992; Field et al., 2012). As a measure of effect size, we report the generalized eta square, η_G_^2^, for comparability across between-subjects and within-subjects designs (Bakeman, 2005). We analyzed the pairwise linear relationships between the components using Spearman's correlation coefficients.

### Results and Discussion

**Figure 1:**
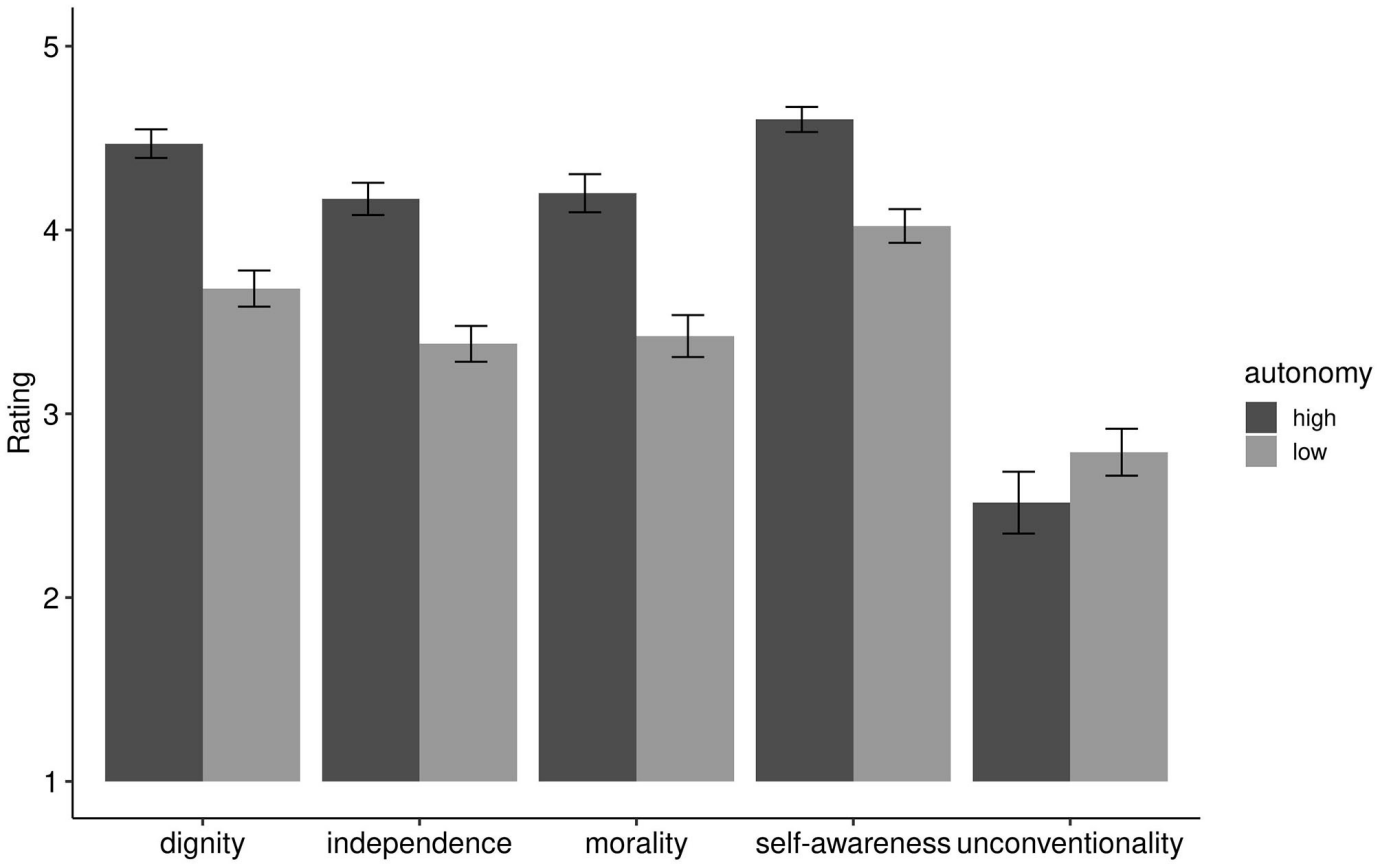
Mean ratings of five components of autonomy at two levels of autonomy. *N* = 175. Rating scales ranged from 1 to 5. Error bars show 95% confidence intervals.

Conducting the ANOVA as preregistered, we found a significant main effect of autonomy level, *F*_(1, 174)_ = 441.94, *p* < 0.001, η_G_^2^ = 0.12, and a significant main effect for the components, *F*_(4, 696)_ = 204.44, Huynh–Feldt corrected *p* < 0.001, η_G_^2^ = 0.39. The interaction of autonomy level and the components was also significant, *F*_(4, 696)_ = 110.61, Huynh–Feldt corrected *p* < 0.001, η_G_^2^ = 0.07. Pair-wise Wilcoxon comparisons revealed significantly higher ratings between high-autonomy and low-autonomy for the components' dignity (*Mdn*_*high*_ = 4.6, *Mdn*_*low*_ = 3.8, *W* = 25,336, *p* < 0.01, *ES* = 0.57, large), independence from others (*Mdn*_*high*_ = 4.2, *Mdn*_*low*_ = 3.4, *W* = 25,020, p < 0.01, *ES* = 0.55, large), morality (*Mdn*_*high*_ = 4.2, *Mdn*_*low*_ = 3.4, *W* = 23,954, *p* < 0.01, *ES* = 0.49, moderate), and self-awareness (*Mdn*_*high*_ = 4.8, *Mdn*_*low*_ = 4.0, *W* = 23,614, *p* < 0.01, *ES* = 0.47, moderate), but not for unconventionality (*Mdn*_*high*_ = 2.6, *Mdn*_*low*_ = 2.8, *W* = 12,668, *p* = 0.005, *ES* = 0.15, small), where the high-autonomy items actually obtained significantly lower ratings compared to the low-autonomy items (see Figure 1). As expected, we found medium-sized correlations between dignity, self-awareness, independence from others, and morality, but not for unconventionality (Table 2).

**Table 2:** Descriptive statistics and Spearman's rank inter-correlations *r*_S_ (*p*-value) for the five autonomy components.

	***M***	***SD***	**1**	**2**	**3**	**4**
1. Dignity	4.08	0.53	–			
2. Independence from others	3.78	0.55	0.50 (<0.01)	–		
3. Morality	3.81	0.66	0.61 (<0.01)	0.33 (<0.01)	–	
4. Self-awareness	4.31	0.47	0.60 (<0.01)	0.39 (<0.01)	0.46 (<0.01)	–
5. Unconventionality	2.65	0.94	−0.05 (0.54)	−0.10 (0.21)	−0.08 (0.32)	−0.11 (0.16)

*N = 175, Holm-Bonferroni correction results in an significance level of α = 0.01 for the p-values*.

## General Discussion

Using a bottom-up empirical approach, we examined laypersons' perceptions of autonomy with components derived from the philosophical and psychological literature. Across three studies, we identified how laypersons exemplify autonomous behaviors. As expected, we found that behaviors characterized by high autonomy are rated significantly higher in their perceived dignity, independence from others, morality, and self-awareness than those low in autonomy. These results show the assumed connection between the scientific perspective on autonomy and the everyday perspective of laypersons, and thereby provide a foundation for further research on the concept of autonomy.

We also found medium-sized correlations between the components' dignity, independence from others, morality, and self-awareness. For the proposed component unconventionality, we did *not* find any significant correlations with the other components, and the effect in the ratings was *reversed*, i.e., the high-autonomy items were rated significantly lower in unconventionality than the low-autonomy items.

Particularly instructive is a qualitative consideration of the sorted items. Looking at the five high-autonomy items, we find two themes. First, the items “choosing a partner,” “staying true to oneself,” and “determining with whom one surrounds oneself with” focus on interpersonal relationships and/or express a clear distinction of the self from others by focusing on oneself. The item “choosing a partner” was rated the highest, suggesting that autonomy especially plays a role in defining one's relationship with other people, and the choice of close ones. The other two high-autonomy items focus on reflected decision-making: “deciding for oneself” and “thinking critically and questioning.” In this manner, high autonomy seems to play a role in both, reflected thinking and deciding as well as in freely determining social relationships.

Conversely, reviewing the five lowest-rated items, we found a wide variety of themes: “acting contrary to societal expectations and laws,” “designing working conditions,” “shaping one's living situation,” “travel,” and “acting uninfluenced by external factors.” On the one hand, “acting uninfluenced by external factors” and “acting contrary to societal expectations and laws” come very close to the definition of autonomy as resistance against external influences (Kohlberg et al., 1983; May, 1994; Erikson, 1998). On the other hand, the social relationship theme in the high-autonomy items suggests that laypersons do not merely see autonomy as a reaction to external influences, but more as a chance to proactively implement their preferences after well-reflected consideration. This entanglement of autonomy with interpersonal factors and reflective thinking has been stressed before (Ryan and Deci, 2006; Chirkov, 2011). It relates to the concept of reflective autonomy proposed by Koestner and Losier (1996), who divide autonomy into *reactive* and *reflective* autonomy. While reactive autonomy is seen as an “interpersonal conception of autonomy that highlights people's desire to resist influence or coercion,” reflective autonomy is a “conception of autonomy that emphasizes people's desire to feel like the origin of their actions and to have input into determining their behavior” (Koestner and Losier, 1996, p. 488). Thus, our findings suggest that laypersons have a view of autonomy that includes both reactive and reflective aspects, but that reflective items (“deciding for oneself,” “thinking critically and questioning,” “choosing a partner,” “staying true to oneself,” and “determining with whom one surrounds oneself with”) appear to weigh on average more heavily than the reactive items (“acting uninfluenced by external factors,” and “acting contrary to societal expectations and laws”). This implication could be further examined in future studies, taking the distinction between reactive and reflective autonomy into consideration.

High-and low-autonomy items were differentiated by the components' dignity, independence from others, morality, and self-awareness. By contrast, the component unconventionality did *not* distinguish between high- and low-autonomy behaviors in the proposed direction but instead showed an unexpected significant *reverse* differentiation. At the same time, the overall average of the ratings was lower for unconventionality than for the other four components, suggesting that this component is generally perceived to be less indicative of autonomy. Contrary not only to the literature but also to our preregistration, unconventionality falls out of line considering the correlations between the components.

However, some examples given in Study 1 did mention unconventionality explicitly, as one person listed “unconventional thinking,” and another participant listed “acting despite conventions.” When we consider the categories, two of the low-autonomy items explicitly name acting “uninfluenced by external influences” and “contrary to societal expectations and laws,” both of which are almost identical to common definitions of unconventionality (Shweder et al., 1990). Notably, however, unconventionality is the only component with an inverted framing (being *not* within conventions), whereas the other components are all positively framed. This may have triggered or at least contributed to the reversal of the difference. Future research should investigate this component using positive phrasing (such as “originality” or “open-mindedness”) congruent with the positive phrasing of the other components.

Another possible explanation may lay in the theoretical foundation of unconventionality as a component of autonomy. This component has been derived from the theory of moral development (Kohlberg, 1981; Kohlberg et al., 1983), where the post-conventional level is the highest level of moral development. According to Kohlberg, however, only a very small number of individuals reach this level, so it is plausible that relatively many participants do not recognize post-conventional behavior as particularly autonomous.

Finally, autonomy is sometimes seen as an equivalent or even synonym of individualism, a view that has been criticized by modern scholars who propose autonomy to be universal (Chirkov, 2008). Our participants' top autonomy items indicate that they perceive autonomy as being “in control” in social relationships, i.e., being able to oppose obligations arising from social relationships (Walter and Ross, 2014). In their (Western) cultural view, the individualistic understanding of autonomy is conventional, and any collectivist, relational, or embedding perspectives are unconventional (Inglehart and Oyserman, 2004). Whether this explains our observation of a (reverse) effect of autonomy on the unconventionality ratings will have to be tested in cross-cultural studies. In light of this, the question of whether, and in what cultural contexts, autonomy leads to greater wellbeing may be addressed (Chirkov, 2008; Walter and Ross, 2014).

Several benefits and insights arise from our findings. Practically, knowing about laypersons' understanding of autonomy could aid psychological research in operationalizing autonomy in scales, surveys, and experiments. Thus, when creating scales to measure autonomy, future research can benefit from taking not only the confirmed components but also the specific examples we collected into account.

At the theoretical level, establishing *self-awareness* as a component of autonomy is in line with the feminist approach to *autonomy-connectedness* (Bekker, 1993; Bekker and van Assen, 2008; Bachrach et al., 2013), which defines self-awareness, next to sensitivity to others and capacity for managing new situations, as one of three sub-scales. The conception of autonomy-connectedness arises from the idea of gender differentiation. It integrates the presumed feminine aspects of identity, including the need and capacity for intimacy and functioning in intimate relationships, and the (more masculine) need and capacity for separation and independence (Bekker, 1993).

Additionally, confirming *morality* as a component is in line with the related constructs of moral agency (Black, 2016) and moral integrity (Arvanitis and Kalliris, 2020). Viewing *dignity* as another component of autonomy can be particularly relevant in the context of health care and nursing. Specifically, it could be helpful for research on and work in geriatric psychology (Randers and Mattiasson, 2004), where fostering the autonomy of patients could lead to more wellbeing and maintaining a sense of dignity. Lastly, the component *independence from others* was also named several times as an example by the laypersons in Study 1. This is in line with the formula autonomy = authenticity + independence (Dworkin, 1988). It is also found in the personality theory by Angyal (1941), proposing that life follows a process between two forces: autonomy as “tendency of the personality toward a greater self-determination” and homonomy as a “tendency toward conformity with the superindividual wholes of society, culture” (Angyal, 1941, p. 365). This demonstrates that autonomy largely depends on the interplay between an individual and their environment, and that an understanding of autonomy as mere independence from others fails to understand the human nature of social beings.

Modern and feminist views on autonomy in particular, e.g., the autonomy-connectedness conception, stress the role of social identity, social interaction, and interdependence instead of independence (Bekker and van Assen, 2008; Pianca and Santucci, 2022). Other feminist authors highlight the need for independence in the sense of objectivity, meaning informed, flexible, and critical attachment to others while considering one's biography and interpretations (Cooke, 1999). Sayer (2011) understands autonomy as self-rule and capacity within social relationships and responsibilities more than as complete independence from others. The author also states that responsibilities are the key to exercising self-command whilst being accountable for others. This relates to empirical studies showing how attachment or interdependence can lead to greater autonomy. According to Collins and Feeney (2004, p. 173), securely attached individuals “are able to maintain close relationships without losing personal autonomy.”

Thus, modern views link autonomy to interdependence rather than independence. Within the framework of SDT, autonomy is defined as self-governance, or rule by the self, whereas heteronomy is defined as the opposite, meaning “regulation from outside the phenomenal self, by forces experienced as alien or pressuring, be they inner impulses or demands, or external contingencies of reward and punishment” (Ryan and Deci, 2006, p. 1562). In noting that individuals may have chosen to be dependent or, conversely, may have been forced into independence due to circumstances, SDT also explicitly distinguishes autonomy from independence. Ryan et al. (2005) state that, while autonomy is commonly equated with independence, SDT differentiates the two by defining dependence strictly in the sense of reliance and finding that people are more likely to depend on those who support their autonomy. In line with the older understanding of independence by classical developmental psychology, we still used independence in this study, and the laypersons found that the high autonomy examples could be differentiated from the low autonomy examples by their independence from others. Nonetheless, future research should take the enhancement from independence to interdependence into account and examine laypersons' ratings of autonomous examples while distinguishing between independence and interdependence.

Strengthening the definition and understanding of autonomy can not only benefit the empirical discourse but may also have an empowering impact on human and societal life through applications. Without a doubt, autonomy is highly important on an individual level, e.g., according to SDT, autonomy is one of the three basic psychological needs for wellbeing (Yu et al., 2018; Ryan and Deci, 2020). Empirically, it has been shown that experiencing higher autonomy without necessarily eliminating extrinsic motivation fosters wellbeing (Kukita et al., 2022). Dignity, independence from others, morality, and self-awareness, may be used, perhaps in a context-specific manner, to specify and enrich practice-oriented discussions and interventions. One example is artificial intelligence (Calvo et al., 2020), but therapeutic or coaching settings are just as plausible, especially considering personality disorders like avoidant personalities. At the workplace, autonomy plays a crucial role in employee engagement and wellbeing (Gagné and Bhave, 2011), where workshops could help to boost self-awareness and autonomous decision-making. In general, our results could improve communication of scientific perspectives in applied settings and also with the public.

The methodological appeal of the approach used in this research is the change from the scientific perspective to the layperson's perspective, which is indicative of everyday relevance and parlance (Kraft-Todd and Rand, 2019). However, it comes with some limitations: First, in the present implementation, following Kraft-Todd and Rand (2019), we used only 10 examples out of the 34 categories. These 10 varied in their level of autonomy, but even the low-autonomy items obtained mean ratings above 2.5, which is the midpoint of the used rating scale. In future investigations, a wider range of autonomy items could be used to compare items that are absolutely high in autonomy to those that are absolutely low.

Additionally, the present research is, even though preregistered, an exploratory investigation, and just as for the research on heroism by Kraft-Todd and Rand (2019), further replications and confirmatory studies are needed. Another shared aspect with the research of heroism by Kraft-Todd and Rand (2019) is that many of the examples, rendered by the lay persons in Study 1, described not so many specific acts but goals, values, and process features underlying mere classes of behaviors (like “deciding what is good for me” or “free voting rights”), even though our instructions explicitly referred to “examples of behaviors.” On the one hand, the relatively high educational level of our participants may partly explain why abstract terms were provided so readily. On the other hand, the over-inclusive and generalizing interpretations that our participants applied to the task instructions may demonstrate how hard it is to break down autonomy (and self-determination) into observable behaviors. For experimenters, this implies that autonomy is difficult to operationalize. Owing to its multi-component and principled nature, the feeling of autonomy appears to be based more on subjective reflections on the antecedents and conditions of choices and preferences than on specific observable and executable behaviors.

Methodologically, since we recruited mainly *via* social media and personal contacts, the samples in all three studies show some selection biases: first, the overall education of our three samples is rather high in comparison to the average population, while their age is lower than representative. Second, more females than males participated in our uncompensated questionnaires. In Study 1, the imbalance amounts to about 2:1 (64% female, 32% male, 2% diverse, 2% not specified). Naturally, our approach builds upon the examples generated in Study 1, and thus the characteristics of the sample of participants generating these. However, in Study 2, where the gender ratio was quite strongly divergent from representative (77% female, 22% male, 0% diverse, 1% not specified), we looked directly into the effects of gender (Table 1), and could not find any practically relevant differences. Moreover, in Study 3, where we had unintentionally exceeded our preregistered sample size, 357 women and, with a ratio of 5:1, only 86 men participated originally. When we paired the male participants with the first 88 female participants to generate a sample with a balanced gender ratio, the resulting pattern was the same in the entire sample of all 444 participants. Taken together, these observations lead us to conclude that gender differences are not a relevant factor in the present results. We do, however, acknowledge an educational bias that is probably related to the distribution of ages. The sample in Study 1 was rather highly educated (49% had college/university degrees) and the age was, even though ranging from 18 to 82, younger than the German average population, with an average of 44 years (Statistisches Bundesamt, 2020). These characteristics might have influenced the choice of youth-specific topics like “choosing a profession” and “deciding for oneself.” Topics that are more relevant to older adults might be underrepresented. To embrace the perspective of patients and older adults (Sherwin and Winsby, 2011), it would be beneficial to include older adult participants in future studies. Finally, our German sample reflects only a small fraction of possible cultural backgrounds and further contributes to the bias of Western, educated, industrialized, rich, and democratic (WEIRD) societies in the social sciences (Henrich et al., 2010). Other cultures, especially those high on collectivism, would certainly show a different understanding and evaluation of autonomy, perhaps one that involves unconventionality to a higher degree. Especially since autonomy is a concept highly valued in individualistic societies, a comparison between more individualistic and more collectivist socialization could allow a more holistic and less WEIRD view of autonomy.

To conclude, the present research helps to characterize the components defining autonomy. We demonstrate an empirical approach to relating scholarly conceptions of autonomy to everyday manifestations. In this sense, our findings delineate the real-life behavioral implications of autonomy.

## Data Availability Statement

The datasets presented in this study can be found in online repositories. The names of the repository/repositories and accession number(s) can be found at: https://osf.io/ugk3w/.

## Ethics Statement

The studies involving human participants were reviewed and approved by Ethics Committee of faculty 05 Goethe University. The participants provided their written informed consent to participate in this study.

## Author Contributions

EZ and SW: conceptualization, data analysis, and writing of the manuscript. EZ: survey construction, data curation, and data visualization. Both authors have read and agreed to the published version of the manuscript. Both authors contributed to the article and approved the submitted version.

## Conflict of Interest

The authors declare that the research was conducted in the absence of any commercial or financial relationships that could be construed as a potential conflict of interest.

## Publisher's Note

All claims expressed in this article are solely those of the authors and do not necessarily represent those of their affiliated organizations, or those of the publisher, the editors and the reviewers. Any product that may be evaluated in this article, or claim that may be made by its manufacturer, is not guaranteed or endorsed by the publisher.
