# Supplementary material for: Grassroots Autonomy: A Laypersons' Perspective on Autonomy
Source: Front Psychol. 2022 Apr 7;13:871797. doi: 10.3389/fpsyg.2022.871797 (PMC9021446; doi:10.3389/fpsyg.2022.871797)
Supplement: Supplementary file 1 [file Data_Sheet_1.pdf]

SUPPLEMENTARY MATERIAL

Supplementary data analysis for Study 3 using *N* = 444

The following shows the analysis using the entire sample of *N* = 444 participants (age: *M* = 39.60, *SD* = 10.15, ranging from 20 years to 75 years; education: 52.93% university degree or college, 19.82% trained profession, 19.81% A-levels, 6.76% secondary school certificate, 0.90% school leaving certificate and 0.23% no finished degree; gender identification: 357 female, 86 male, 1 diverse). Mean participation time was *M* = 7.31 minutes. Materials, Procedures, and Statistical Analysis were the same as described in the in the main article for the *N* = 175 sample.

Results and Discussion show the same result pattern as for the *N* = 175 sample: Conducting the ANOVA, as preregistered, we found a significant main effect of autonomy level, $F_{(1,443)}$ = 1296.83, $p < .001$, $\eta_G^2 = 0.11$, and a significant main effect of the components, $F_{(4,1772)}$ = 521.16, Hynh-Feldt corrected $p < .001$, $\eta_G^2 = 0.40$. The interaction of autonomy level and the components was also significant, $F_{(4,1772)}$ = 270.82, Hynh-Feldt corrected $p < .001$, $\eta_G^2 = 0.07$). Pair-wise Wilcoxon comparisons revealed significantly higher ratings for high-autonomy and low-autonomy items for the components' dignity (Mdn_{high} = 4.6, Mdn_{low} = 3.8, *W* = 158860, $p < .01$, *ES* = 0.53, large), independence from others (Mdn_{high} = 4.2, Mdn_{low} = 3.4, *W* = 163766, $p < .01$, *ES* = 0.57, large), morality (Mdn_{high} = 4.4, Mdn_{low} = 3.4, *W* = 154536, $p < .01$, *ES* = 0.49, moderate), and self-awareness (Mdn_{high} = 4.8, Mdn_{low} = 4.2, *W* = 149806, $p < .01$, *ES* = 0.46, moderate), but not for unconventionality (Mdn_{high} = 2.4, Mdn_{low} = 2.8, *W* = 83826, $p < .01$, *ES* = 0.13, small), were the high-autonomy items actually obtained significantly lower ratings compared to the low-autonomy items (see **Supplementary Figure 1**). As expected, we found medium-sized correlations between dignity, self-awareness, independence from others, and morality, but not for unconventionality (**Supplementary Table 1**). The size of the inter-correlations varies slightly between the analyses using *N* = 444 and *N* = 175.

SUPPLEMENTARY FIGURE 1 | Mean ratings of five components of autonomy at two levels of autonomy. *N* = 444. Rating scales ranged from 1 to 5. Error bars show 95% confidence intervals.

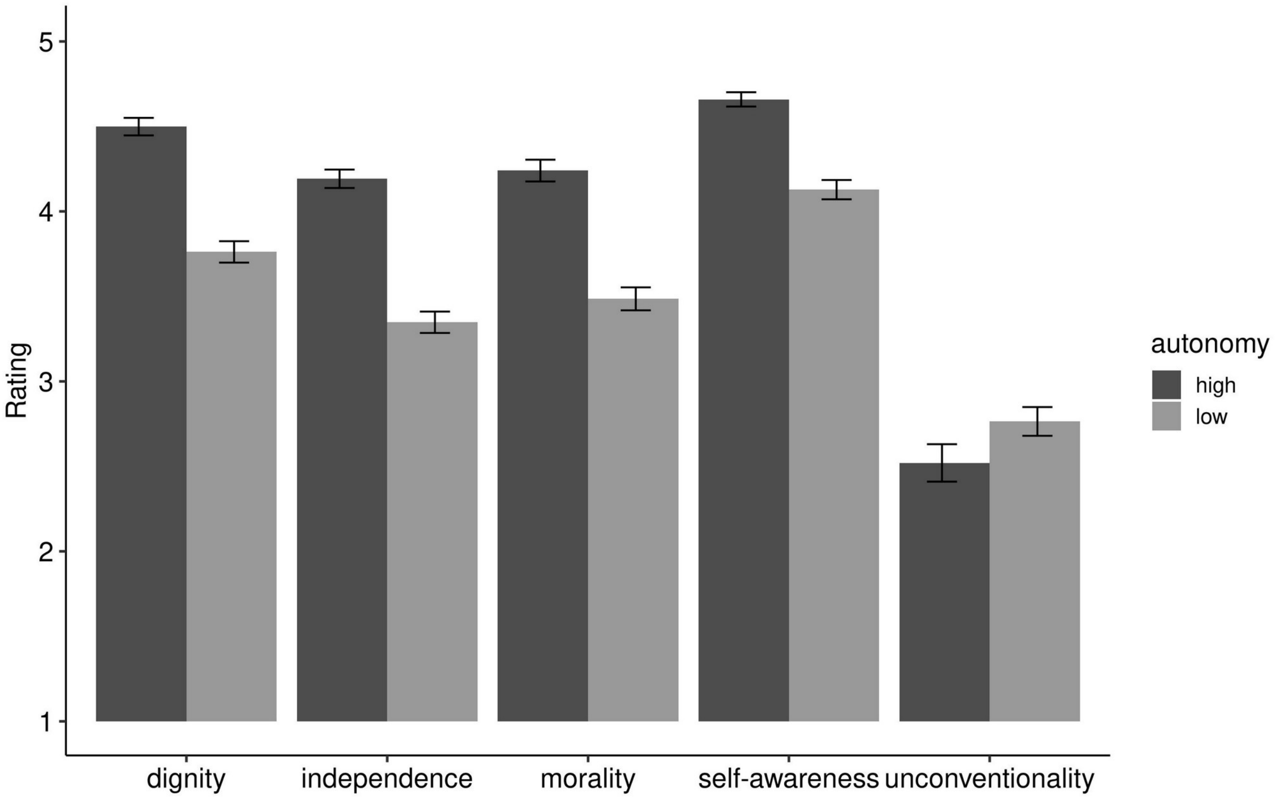

SUPPLEMENTARY TABLE 1 | Descriptive statistics and Spearman's rank inter-correlations r_S (*p* - value) for the five autonomy components

	M	***SD***	**1**	**2**	**3**	**4**
1. dignity	4.13	0.55	–			
2. independence from others	3.77	0.56	.37 (< .01)	–		
3. morality	3.86	0.64	.58 (< .01)	.31 (< .01)	–	
4. self-awareness	4.39	0.47	.51(< .01)	.27 (< .01)	.37 (< .01)	–
5. unconventionality	2.64	1.00	-.08 (.10)	-.06 (.22)	-0.06 (.22)	-.04 (.35)

N = 444, Holm-Bonferroni correction results in a significance level of α = .01 for the *p*-values.
